# Principle, application and challenges of development siRNA-based therapeutics against bacterial and viral infections: a comprehensive review

**DOI:** 10.3389/fmicb.2024.1393646

**Published:** 2024-06-13

**Authors:** Hamid Motamedi, Marzie Mahdizade Ari, Amirhoushang Alvandi, Ramin Abiri

**Affiliations:** ^1^Student Research Committee, School of Medicine, Kermanshah University of Medical Sciences, Kermanshah, Iran; ^2^Department of Microbiology, School of Medicine, Kermanshah University of Medical Sciences, Kermanshah, Iran; ^3^Department of Microbiology, School of Medicine, Iran University of Medical Sciences, Tehran, Iran; ^4^Microbial Biotechnology Research Center, Iran University of Medical Sciences, Tehran, Iran; ^5^Medical Technology Research Center, Health Technology Institute, Kermanshah University of Medical Sciences, Kermanshah, Iran; ^6^Fertility and Infertility Research Center, Health Technology Institute, Kermanshah University of Medical Sciences, Kermanshah, Iran

**Keywords:** RNAi, siRNA, non-coding RNA, delivery systems, therapeutic applications

## Abstract

While significant progress has been made in understanding and applying gene silencing mechanisms and the treatment of human diseases, there have been still several obstacles in therapeutic use. For the first time, ONPATTRO, as the first small interfering RNA (siRNA) based drug was invented in 2018 for treatment of hTTR with polyneuropathy. Additionally, four other siRNA based drugs naming Givosiran, Inclisiran, Lumasiran, and Vutrisiran have been approved by the US Food and Drug Administration and the European Medicines Agency for clinical use by hitherto. In this review, we have discussed the key and promising advances in the development of siRNA-based drugs in preclinical and clinical stages, the impact of these molecules in bacterial and viral infection diseases, delivery system issues, the impact of administration methods, limitations of siRNA application and how to overcome them and a glimpse into future developments.

## 1 Introduction

**FIGURE 1:**
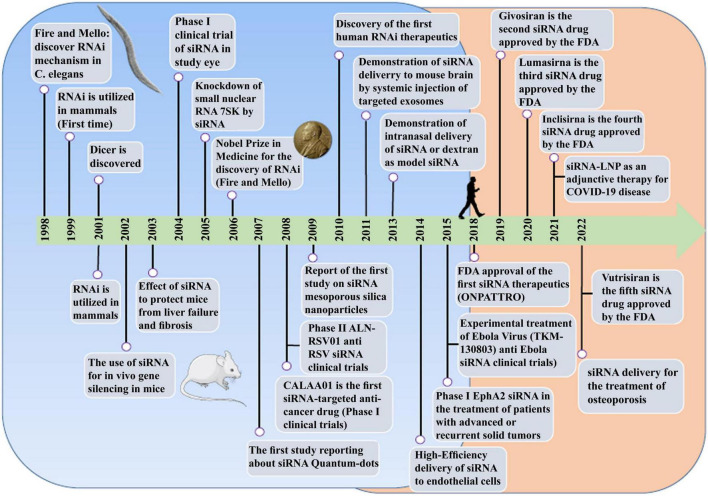
The comprehensive event of the discovery and elucidation of the RNAi pathway.

RNA therapy refers to the treatment or prevention of diseases using RNA-based molecules. The first siRNA-based treatment was approved by the Food and Drug Administration (FDA) in 2018 (Figure 1; Kim et al., 2019; Kim, 2022). Since the advent of RNA interference (RNAi), significant progressions has been made in understanding and applying gene silencing mechanisms and treating human diseases (Saw and Song, 2020). Different classes of small RNAs are categorized based on various aspects like the origin, structure, related effector proteins, and biological roles. Based on the coding potential, RNA molecules are categorized into coding RNAs and noncoding RNAs (ncRNAs). ncRNAs are a group of RNAs that do not encode functional proteins, including long noncoding RNAs (lncRNAs), small interfering RNAs (siRNAs), microRNAs (miRNAs), and piwi interactors (piRNAs) are known. SiRNAs, microRNAs, and piRNAs exist only in eukaryotes cells. However, Argonaute proteins, in addition to their eukaryotic silencing function, can also be found in scattered bacterial and archaeal species (Ghildiyal and Zamore, 2009; Kaikkonen et al., 2011). SiRNAs and miRNAs are the most widely distributed phylogenetically. The precursors of these two molecules are double-stranded and both of them can be considered as targets for the treatment of many different diseases, including cancers (Bader et al., 2011; Tabernero et al., 2013; Schultheis et al., 2014), and infections (DeVincenzo et al., 2010; Chandra et al., 2012; Tribolet et al., 2020). On the other hand, piRNAs are animal specific small silencing RNAs. Unlike siRNAs (20–23 nucleotide RNA duplex with 2 nucleotides 3′overhang) and miRNAs (19–25 nucleotide RNA duplex with 2 nucleotides 3′overhang), piRNAs are processed from long single-stranded (24–30 nt) precursor transcripts and do not require Dicer enzyme for processing (Cai et al., 2022). In addition, piRNAs have 2′-O-methyl modified 3′ and direct PIWI-clade proteins. But siRNAs and miRNAs bind to members of the Ago clade of Argonaute proteins (Faehnle and Joshua-Tor, 2007).

Small interfering RNA molecules are potential therapeutic agents for a wide range of diseases, including cancer, viral and bacterial infections (Yanagihara et al., 2006; Lares et al., 2010). SiRNAs can protect the cells by inhibiting the replication of viral agents like human immunodeficiency virus (HIV), influenza virus (INFV), hepatitis B virus (HBV), hepatitis C virus (HCV), SARS coronavirus (SARS−CoV), human papillomavirus (HPV), and West Nile virus (Tan and Yin, 2004; Qureshi et al., 2018). Accordingly, it seems that RNAi-based drugs are suitable options for the treatment of several severe viral infections (Qureshi et al., 2018),but more studies are needed for the use of these molecules as preventable vaccines for viral infections. On the other hand, siRNAs have inhibited the expression of genes in bacterial infections (Yanagihara et al., 2006; Zhang et al., 2007; Xu et al., 2009). Also, bacteria can be used as carriers to transfer silencing genes. For example, attenuated *Salmonella enterica* serovar typhimurium (*Salmonella typhimurium*) was used as a vectors to deliver hairpin silencing RNA (shRNA) expression plasmids to mammalian cells in 2007. This approach led to gene silencing *in vitro* (cancer cell lines) and *in vivo* (mice containing implanted tumors) (Zhang et al., 2007).

The potential application of siRNAs as a probable therapeutic is being investigated in several clinical trials (Setten et al., 2019). Since 2004 until now, when the first clinical trial targeting siRNA was introduced by intravitreal injection in patients with age-related macular degeneration (AMD), extensive research has been conducted on the application of siRNA in therapy. The clinical trials were performed to investigate the effects of siRNA to treat diseases like non-arterial anterior ischemic optic neuropathy (Solano et al., 2014), delayed graft function (Lam et al., 2012; Peddi et al., 2014), transthyretin-mediated amyloidosis (Coelho et al., 2013; Suanprasert et al., 2014), cancer (Davis et al., 2010; Tabernero et al., 2013), hepatitis B (Wooddell et al., 2013), Ebola (Davis et al., 2010), hypercholesterolemia (Fitzgerald et al., 2014), diabetic macular edema (Lee et al., 2012), etc. Interestingly five siRNA-based drugs (Patisiran, Givosiran, Inclisiran, Lumasiran, and Vutrisiran) have been approved by the FDA (Table 1) and several potent drugs are in the final stages of phase III clinical trials (Table 2; Friedrich and Aigner, 2022).

**TABLE 1:** Smallinterfering RNA drugs approved by the FDA.

Therapeutic drug name	Disease	Target	Delivery	Route of administration	Target organ
Patisiran (Onpattro)	Transthyretin-mediated amyloidosis	TTR	Lipid nanoparticle (DLin-MC3-DMA)	IV	Liver
Givosiran (Givlaari)	Acute hepatic porphyria	ALAS-1	GalNAc conjugate	SC	Liver
Inclisiran (Leqvio)	Hypercholesterolemia	PCSK9	GalNAc conjugate	SC	Liver
Vutrisiran (Amvuttra)	Transthyretin-mediated amyloidosis	TTR	GalNAc conjugate	SC	Liver
Lumasiran (Oxlumo)	Primary hyperoxaluria type 1	HAO1	GalNAc conjugate	SC	Liver

TTR, transthyretin; ALAS-1, aminolevulinate synthase 1; PCSK9, proprotein convertase subtilisin kexin type 9; HAO1, hydroxyacid oxidase 1.

**TABLE 2:** Small interfering RNA-based drugs in the final stages of phase III clinical trials.

Therapeutic drug name	Disease	Target	Delivery	Route of administration	Clinical trial number	Clinical trial stage	Status
Fitusiran (ALN-AT3SC)	Hemophilia A/B	Antithrombin mRNA	GalNAc	SC	NCT03974113	Phase III	Active, not recruiting
Nedosiran	Primary hyperoxaluria	Hepatic lactate dehydrogenase mRNA	GalNAc	SC	NCT04042402	Phase III	Enrolling by invitation
Teprasiran	Cardiac surgery	p53 mRNA	–	IV	NCT02610296	Phase III	Completed
Cosdosiran	Primary angle-closure glaucoma	Caspase 2	–	IV	NCT02341560	Phase II/III	Terminated
Tivanisiran	Dry eye disease	Transient receptor potential cation channel subfamily V member 1 (TRPV1) mRNA	–	Topical eye drop	NCT03108664	Phase III	Completed

In this review, we have discussed the key and promising advances in the development of siRNA-based drugs in preclinical and clinical stages, the impact of these molecules in bacterial and viral infection diseases, delivery system issues, the impact of administration methods, limitations of siRNA application and how to overcome them and a glimpse into future developments.

## 2 Non-coding RNA

**FIGURE 2:**
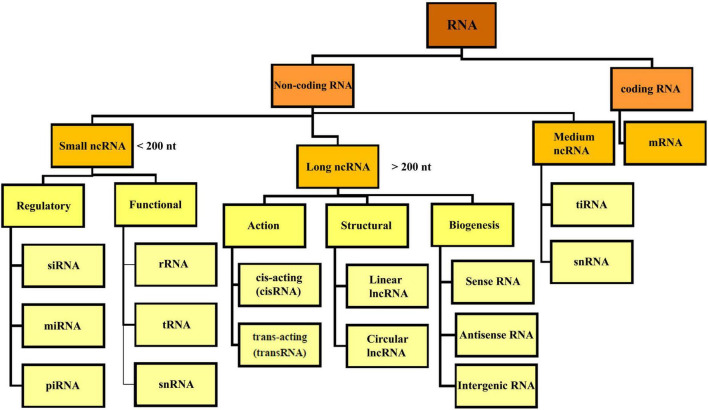
Overview classification of RNA. There are different types of RNA in the cell which are divided into coding and non-coding RNA. NcRNA, non-coding RNA; siRNA, small interfering RNA; miRNA, micro-RNA; piRNA, piwi interacting RNA; rRNA, ribosomal RNA; mRNA, messenger RNA; tRNA, transfer RNA; ciRNA, circular intronic RNA; snRNA, small nuclear RNA; tiRNA, tRNA-derived stress-induced RNA.

Based on the coding potential, RNA molecules are categorized into coding RNAs and ncRNAs (Figure 2). NcRNAs were initially identified in 1973 as functional non-coding transcripts that do not encode any proteins (Li and Liu, 2019). The regulatory functions of the ncRNAs in eukaryotic and prokaryotic cells were published in the 1980s (Arraiano, 2021). The mechanisms of gene regulation of ncRNAs were recognized in 2002. Considering the nucleotide size, ncRNAs are categorized to small ncRNAs (less than 200 nt) and long ncRNAs (lncRNAs, more than 200 nt). Small ncRNAs classified into the housekeeping ncRNAs like transfer RNA (tRNA), ribosomal RNA (rRNA), small nuclear RNA (snRNA), and regulatory ncRNAs like miRNAs, siRNA, and circular RNAs (circRNAs). LncRNAs are categorized based on their structure (linear and circular), area of activity (sense, anti-sense, intronic, and intergenic), and mechanism of action (transRNA, cisRNA and CeRNA) (Delihas, 2015; Bhatti et al., 2021; Winkle et al., 2021). lncRNAs consists of stand-alone lncRNAs, natural antisense transcripts, pseudogenes, and abundant short transcripts (Kung et al., 2013). circRNAs are another type of ncRNAs that have a circular structure that makes them resistant to ribonucleases (Arraiano, 2021).

MicroRNA was discovered by Victor Ambros in 1993 during his research on lin-4 gene of *Caenorhabditis elegans* (Lee et al., 1993). MiRNAs are endogenous 17–25 nucleotides single-stranded molecules which are applied for diagnostic purposes and treatment of diseases like lung adenocarcinoma, prostate, gastric, breast, liver, and colorectal cancers (Moran et al., 2014; Arraiano, 2021; Ho et al., 2022). Following the synthesis of long miRNAs by RNA polymerases, pri-miRNAs are produced by ribonucleases in the nucleus and converted into pre-miRNAs with a loop structure. Pre-miRNAs are transferred from the nucleus to the cytosol by RanGTP-dependent double-stranded RNA (dsRNA)-binding proteins like Exportin 5. The pre-miRNAs turn to mature double-stranded miRNA by the RNase III Dicer. MiRNAs accompanied by RNA helicase and the RISC protein complex can perform the task as a gene regulator complex (Winkle et al., 2021). There are potential drugs like RG-012 and MGN-2677 targeting miR-122 and miR-143/145 for the treatment of nephropathy and vascular disease which are under investigation in different clinical trial stages. MRX34 and Remlarsen targeting miR-34a and miR-29 are in phase I clinical trial to study cervical, ovarian and colon cancer, and cutaneous and pulmonary fibrosis, respectively. Currently, Miravirsen targeting miR-122 is being investigated in phase II clinical trial for the treatment of HCV (Chakraborty et al., 2020).

Long noncoding RNAs were first discovered in the 1990s. The biogenesis of lncRNAs are similar to miRNAs, but they are longer about 200 nucleotides (Cabili et al., 2011; Kung et al., 2013). LncRNAs consist of two main parts; the interactor which is important in the interaction with lipid, protein and nucleic acids, and the structural elements that create the secondary and tertiary structures. Such interactions in secondary structures make the lncRNAs more functional compared to miRNAs. lncRNAs have a role in the gene expression regulating, transcriptional and post-transcriptional regulation (Zhu et al., 2013). In addition to regulating gene expression, they contribute in biological pathways such as the immune (Amit et al., 2020) and the nervous system (Andersen and Lim, 2018). Different lncRNAs can have vital roles in regulation of transcription (e.g., TARID, APOLO, and ANRIL), post-transcriptional regulation (PNCTR, PNUTS, TINCR, and TINCR), cellular organelles (RMRP and SAMMSON), structural functions (NEAT1 and MALAT1), and genome integrity (GUARDIN, lincRNA-p21, and DINO) (Statello et al., 2021). LncRNAs based therapy is a promising method to treat various diseases and cancers and several cardiovascular diseases, e.g., heart failure and hypertension, neurological diseases such as Alzheimer, Parkinson, and metabolic disorders like diabetes (Chen Y. et al., 2021). LncRNAs also regulate viral life cycle, viral gene expression and their pathogenesis (Li et al., 2022). Triggering of the immune system leads to the activation of host lncRNAs which are involved in the antiviral response by stimulating the secretion of interferons (IFN-1), so it can be proposed for treatment of coronavirus disease 2019 (COVID-19) (Guttman et al., 2009; Peng et al., 2010). AC009088, LINC02384, AL392172, and HOTAIRM1 are examples of lncRNAs involved in the regulation of COVID-19 by downregulation Pycard, regulating IFN-γ, and IL-17 signaling pathway, respectively (Moazzam-Jazi et al., 2021; Yang et al., 2021). CRISPR–Cas9, siRNAs, antisense oligonucleotides (ASOs), ASO anti-microRNAs (antimiRs), shRNAs, and circRNAs are a number of RNA based therapies (Ling et al., 2013; Rupaimoole and Slack, 2017) which among the aforementioned molecules, siRNAs or ASOs based therapies are approved and licensed by FDA (Winkle et al., 2021). LncRNAs therapeutic application for preeclampsia (PE)^1^ and lung cancer^2^ are being investigated in different clinical trials.

Small interfering RNA showed successful inhibitory effects on tumor growth, high specificity, low adverse effects, cost-effectiveness, safety, and high efficiency at very low doses. Influencing factor on the efficiency outcomes of siRNA are including as the availability of target sequence on mRNA, stability and structural characteristics of siRNA. In contrast to antisense agents, siRNAs are resistant against nuclease enzymes degradation (Li et al., 2006). In comparison to monoclonal antibody-based drugs, siRNA recognizes its target through pairing the Watson–Crick pattern with mRNA (Hu et al., 2020). As specified by Gupta et al. (2019), the safety of siRNA refers to the fact that there are no chemicals and dangerous materials used in siRNA synthesis processes. Moreover, in contrast to other antisense agents, mode of action of siRNA and their inhibitory effects on gene expressions is carried out in post-translation stages without interfering with DNA or induction of any mutation in its structure (Xu and Wang, 2015; Subhan and Torchilin, 2020). Also, siRNA synthesis is cost-effective because it does not require expensive complex tools. They can undergo changes by some modifications which can confer beneficial effects on their stability in serum and inhibitory efficacy (Braasch et al., 2003; Chiu and Rana, 2003).

## 3 Mechanism of action of RNAi

**FIGURE 3:**
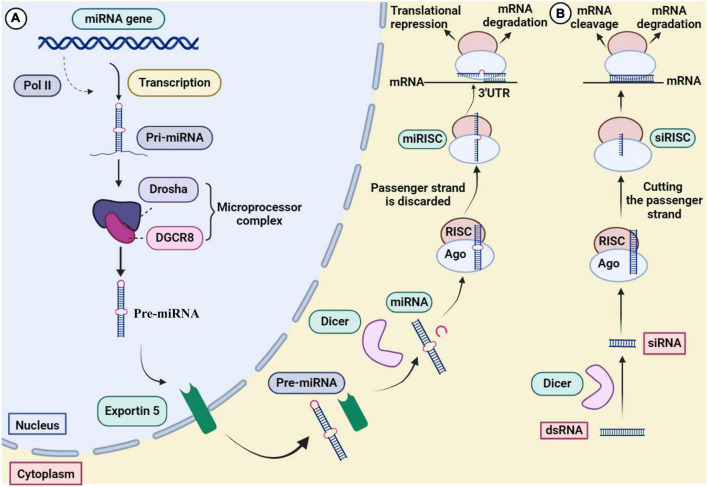
Mechanism of gene silencing through **(A)** miRNA and **(B)** siRNA.

RNA interference has evolved to control gene expression in various organisms (Robb et al., 2005). The mechanism of miRNA and siRNA gene silencing at the post-transcriptional level is depicted in Figure 3. Although some structural similarities are observed in the molecules, the mechanism of action and clinical applications are different (Lam et al., 2015). MiRNAs are small non-coding RNAs (18–24 nucleotides) that regulate gene expression at the translational level (Kobayashi and Singer, 2022). MiRNA biogenesis initially begins by processing from precursor molecules (primiRNA) in the nucleus. The primiRNA are either transcribed from independent miRNA genes or are part of introns of protein-coding RNA polymerase II transcripts. Pri-miRNAs are folded into hairpin structures and further processed by RNA binding protein DiGeorge syndrome critical region gene 8 (DGCR8) and RNase III type endonucleases Drosha (Filipowicz et al., 2008; O’Brien et al., 2018). DGCR8/Pasha is an essential cofactor for Drosha which has been identified in *Drosophila melanogaster* (Wu et al., 2012). Unlike Drosha, DGCR8 directly and stably interacts with pri-miRNAs. Microprocessor complex (Drosha-DGCR8) processes pri-miRNAs to about 70-nucleotide hairpins known as pre-miRNAs. Then, the pre-miRNA located in the nucleus is transported to the cytoplasm by Exportin 5 and further cleaved by Dicer (RNase III endonuclease) to generate the 18–25 nucleotide miRNA duplex. The Dicer enzyme cleaves the terminal loop and finally an RNA-induced silencing complex (RISC)-loading is formed with Argonaute protein (Ago1–Ago4). MiRNA duplex associates with RISC and forms a complex called miRISC. The miRNA duplex is denatured; the passenger strand is discarded. In siRNA processing, AGO2 cleaves the siRNA passenger strand (Liu et al., 2004). In association with the miRNA pathway, miRISC is directed by the guide strand to the target mRNA by partially complementary binding. In the siRNA pathway, the guide strand (antisense) directs the active RISC to the target mRNA, and full complementary binding between the siRNA guide strand and the target mRNA leads to mRNA cleavage.

## 4 Developments in siRNA therapeutics for cancer

RNA interference manage to switch off the main genes involved in cancer and viral infections (Saraswathy and Gong, 2014; Siegel et al., 2014). Same as viral diseases, siRNAs are effective in the treatment of cancers by blocking malignancy-related genes (Table 3; Elbashir et al., 2001; Debela et al., 2021). SiRNA affect the tumor in three ways: inhibition of angiogenesis, inhibition of tumor survival and induction of apoptosis. The process of angiogenesis and vascular endothelial growth factor receptor (VEGFR-1 and VEGFR-2) as the main glycoprotein involved molecule play role in regulation of the of tumor cells growth, metastasis and preventing the death of vasculature by activating signaling pathways like Ras-MAPK (mitogen-activated protein kinase) pathways (Chen and Huang, 2008). The aforementioned pathways are induced by phosphorylation of VEGFR which then followed by cell migration as the initial step of metastasis. VEGF gene and VEGFR gene targeting siRNAs reduce VEGF expression and receptor blocking, and finally inhibition of angiogenesis (Kou et al., 2005). Proto-oncogenes and oncogenes as the main causes of cell growth induction and/or anti-apoptosis effectors can potentially be inactivated by siRNAs. Also, the inhibitory function of siRNAs in silencing of the Wnt pathway was shown in breast and lung cancers (Rosell et al., 2006; Wieczorek et al., 2008).

**TABLE 3:** Anticancer siRNA in pre-clinical and clinical trials.

Therapeutic drug name	Tumor	Target	Vector	Route of administration	Clinical trial number	Clinical trial stage	Status
Atu027	PDAC	PKN3	–	IV	NCT00938574	I	Completed
siG12D LODER	PDAC	KRAS	–	Intratumoral	NCT01188785	I	Completed
Mesenchymal stromal cells with KRAS G12D	Pancreatic cancer	KrasG12D mutation	NP	IV	NCT03608631	I	Recruiting
TKM-080301	Hepatocellular carcinoma	PLK1	NP	IV	NCT01437007	I	Completed
EPHARNA	Advanced malignant solid neoplasm	EphA2	NP	IV	NCT01591356	I	Recruiting
NU-0129	Gliosarcoma and glioblastoma cancer	BCL2L12	NP	IV	NCT03020017	I	Completed
ALN-VSP02	Solid tumors	VEGF and KSP	NP	IV	NCT01158079	I	Completed
CALAA-01	Solid tumors	RRM2	NP	IV	NCT00689065	I	Terminated
DCR-MYC	Solid tumors	MYC	NP	IV	NCT02314052	II	Terminated
NBF-006	Colorectal, pancreatic, and lung cancer	GSTP	NP	IV	NCT03819387	I	Active, not recruiting
CpG-STAT3 siRNA (CAS3/SS3)	Relapsed/refractory B-cell NHL	TLR9 receptor and STAT3	–	Intratumoral	NCT04995536	I	Recruiting
Proteasome siRNA	Metastatic melanoma	Immunoproteasome beta subunits LMP2, LMP7, and MECL1	–	Intradermal	NCT00672542	I	Completed
APN401	Solid tumors (pancreatic and colorectal cancer)	Blocking of the enzymes needed for cell growth	–	IV	NCT03087591	I	Completed
MiHA-loaded PD-L-silenced DC vaccine	Hematological malignancies	PD-L1/PD-L2	–	IV	NCT02528682	I/II	Completed
SLN124	Polycythemia	TMPRSS6	–	SC	NCT05499013	I/II	Recruiting
DCR-MYC	Hepatocellular carcinoma	MYC	NP	IV	NCT02110563	I	Terminated
Atu027	PDAC	PKN3	NP	IV	NCT01808638	II	Completed
TKM-080301	Hepatocellular carcinoma	PLK1	NP	IV	NCT01262235	II	Completed
TKM-080301	Hepatocellular carcinoma	PLK1	NP	IV	NCT02191878	II	Completed
STP705	isSCC	TGF-β1 and COX-2	–	Intralesional	NCT04844983	II	Active, not recruiting
siG12D LODER	PDAC	KRAS G12D mutation	NP	Locally by surgery	NCT01676259	II	Recruiting
–	Neuroblastoma	B4GALNT3	–	NA	NCT01058798	NA	Completed
–	Neuroblastoma	AHR	–	NA	NCT01075360	NA	Completed
–	Chronic myeloid leukemia	NA	SV40	NA	NCT00257647	NA	Completed
–	Cervico-vaginal cancers and precancerous lesions	E6 and E7	–	Vaginal	NCT04278326	NA	Recruiting

PKN3, protein kinase N3; PDAC, pancreatic ductal; CRPC, metastatic castration-resistant prostate cancer adenocarcinoma; NP, nanoparticles; IV, intravenous; SC, subcutaneous; TMPRSS6, targeting transmembrane protease, serine 6; B4GALNT3, β1,4-N-acetylgalactosaminyltransferase III; AHR, aryl hydrocarbon receptor; isSCC, squamous cell carcinoma; NHL, non-Hodgkin lymphoma; SV40, simian vacuolating virus 40; MiHA, minor histocompatibility antigens; Hsp27, heat shock protein.

Heparin-binding EGF-like growth factor (HB-EGF) is involved in biological processes like skin wound healing, heart and eyelid development, and the formation of malignant tumors through the interaction with the signaling molecules downstream of ErbB receptors. As HB-EGF levels are increasing in cancers, it seems that HB-EGF expression is essential in tumorigenicity. Introducing specific drug to target HB-EGF can inhibit tumor growth. For example, Okamoto et al. (2018) showed a reduction in HB-EGF expression in Breast Cancer cell line using lipid nanoparticles encapsulating siRNA with a Fab′ antibody against heparin-binding EGF-like growth factor (αHB-EGF LNP-siRNA). Recently, it has been found that NF-κB activation is related to malignant cell survival by inhibition of apoptosis-related genes (Guo et al., 2005; Yu et al., 2020). Direct or indirect suppression of NF-κB pathway, increase the sensitivity of cancerous cells to apoptosis. For example, considering the fact that HIF-1α is important in NF-κB activation, Chen et al. investigated the adeno-associated virus carrying siRNA targeting HIF 1α (rAAV-siHIF) to induce apoptosis in pancreatic cancer cells. rAAV-siHIF results in reduction of HIF-1α expression and activation of apoptosis in MiaPaCa2 cells (Miyamoto et al., 2006).

Human telomerase is composed of two components, human telomerase reverse transcriptase (hTERT) and RNA (hTR). Telomerase manage immortality and malignancy development through hTERT. hTERT exist in low levels in healthy cells compared to the same cancer cells. SiRNA targeting hTERT-sensitized cervical cancer cells to radiation therapy by decreasing hTERT mRNA (Wang et al., 2007). HER-2/neu and Bcl-2 as an anti-apoptotic proteins are highly expressed in several cancers like human breast cancer and gastric cancer, respectively. HER-2/neu, as an epidermal growth factor receptor (EGFR) family make cancers resistant to apoptosis and it activates protein kinase B (PKB, or Akt) to phosphorylation and ubiquitination of the mouse double minute 2 (MDM2), which this protein degrades the tumor suppressor p53 protein. MDM2 is mostly found in osteosarcoma, breast and ovarian cancer. Obstructing Bcl-2 and Akt pathway or HER-2/neu by blocking agents like siRNA, decrease Bcl-2 expression in gastric cancer cell, decrease telomerase activity, and increases p53 level which regulate apoptosis and suppress cancer cell growth (Wang et al., 2001; Zhou et al., 2001; Hao et al., 2007; Chen and Huang, 2008).

## 5 Therapeutics siRNA for microbial infections

### 5.1 Bacterial infection

Usually, bacteria are not affected by the silencing action of siRNAs because they do not use host cell replication tools (Lieberman et al., 2003; Leung and Whittaker, 2005; López-Fraga et al., 2009). Some bacterial pathogens like *Mycobacterium tuberculosis* (MTB), *Listeria monocytogenes*, *Mycobacterium fortuitum*, *S. typhimurium*, and *Yersiniaceae* need host cell facilities for entry and invasion (Agaisse et al., 2005). Inactivating the invasion related genes like SEC22A, Rab1B, and VPS33B by siRNA can prevent bacterial cell entrance to the host cells (Menanteau-Ledouble et al., 2020). In our recent *in vitro* study, we designed specific siRNA against urease B subunit (ureB) and cytotoxin-associated gene A (CagA) genes from *Helicobacter pylori*. Both virulence factors play an important role in gastric cancer caused by *H. pylori*. The findings of our study showed that targeting *ureB* and *cagA* genes with siRNA is a new strategy to inhibit urease enzyme activity, reduce inflammation and colonization rate (Motamedi et al., 2023). Menanteau-Ledouble et al., reported that *siRNA* targets and silence zipper and trigger processes related to genes in *Yersinia ruckeri*, the causative agent of enteric red mouth in fish. They reported that *Rab1A*, *myotubularin*, *Lama2*, and *Rac1* were more strongly silenced by siRNA (Criss and Casanova, 2003; Menanteau-Ledouble et al., 2020). Silencing of *Caveolin-2* as a host gene involved in the invasion of *Pseudomonas aeruginosa* by siRNAs leads to the reduction of bacterial pathogenesis (Zaas et al., 2005). SiRNA accelerates the clearance of microorganisms by regulating inflammation (Gong et al., 2014). For example, siRNA can reduce the excessive amount of tumor necrosis factor-α (TNF-α) in inflammatory conditions like sepsis (Sørensen et al., 2003). The genes of glutamine synthetase and β-hexosaminidase, the enzymes involved in Mycobacterium cell wall biosynthesis and peptidoglycan hydrolase activity, respectively, can be considered as potential targets for silencing by siRNA (Harth et al., 2000; Koo et al., 2008). Also, siRNA is a promising tool for combating drug-resistant bacterial infections. Yanagihara et al. (2006) showed that silencing coagulase gene reduce staphylocoagulase gene expression and MRSA count in pulmonary infections. In another study, application of siRNA targeting *MexB* efflux pump gene in *P. aeruginosa* significantly decreased the bacterial load in lung infections (Gong et al., 2014). Bacterial strains like attenuated *Salmonella* are used as vehicles for siRNA delivery to melanoma, cervical, and colorectal cancer cells (Zhao et al., 2019; Chen J. et al., 2021). Combination of chloroquine as an anti-malarial agent and novel anti-cancer drug with an anti-Programmed Death-1 (PD-1) siRNA which carried by recombinant attenuated Salmonella to Colon Cancer, showed a significant reduction in cancer cell survival by induction of apoptosis (Lu et al., 2021). Co-administration of PD-L1 targeting siRNA and lenvatinib can improve the treatment of Hepatocellular (HCC) carcinoma (Chen et al., 2022).

As electrostatic interaction and hydrophobic-hydrophilic balance consider for are considered an antibacterial polycation carrier and optimized gene delivery, Zhang et al. (2022) showed siRNA delivery with (triblock amphiphilic polycation micelles) TDDE-3 micelles confer strong antibacterial activity with low MIC need for *Escherichia coli* and *Staphylococcus aureus*.

### 5.2 Viral infection

In recent years, scientists have widely used RNAi to target a number of viral genes to inhibit their expression as well as therapeutic applications (Leonard and Schaffer, 2006). SiRNAs offer a promising therapeutic strategy to combat viral pathogenesis as these molecules target various genes of lethal viruses such as HBV, HCV, HIV, influenza virus, SARS−CoV, HPV, and WNV in infected cells displayed encouraging results in inhibiting viral replication. We have referred to the clinical trials that have been conducted so far for various human viruses based on the direct effect of siRNA to inhibit viral infections.

#### 5.2.1 Respiratory syncytial virus

Respiratory syncytial virus (RSV) belongs to the Paramyxoviridae family and is a nonsegmented negative-strand enveloped RNA virus that is recognized as a major viral respiratory pathogen (Zhang et al., 2005). RSV is the most common cause of hospitalization in infants and also the main cause of bronchiolitis, otitis media and pneumonia in children (less than 1-year-old) (Zhang et al., 2005; Zhang and Tripp, 2008). Currently, there is no effective vaccine for this virus. The RSV genome contains about 15,200 nucleotides, which encode 11 different proteins, including two non-structural (NS1 and NS2) proteins and nine structural proteins (Zhang et al., 2005). For this reason, the use of siRNA to knock down these proteins is an important point that can play a potential role in the treatment of RSV.

The beginning of research related to the role of siRNA in targeting RSV proteins met with good progress. Meanwhile, in 2001 an almost 90% reduction of phosphoprotein (P) from RSV virus was demonstrated using 10 nM dsRNA (Bitko and Barik, 2001). In 2004, researchers were able to specifically prevent and inhibit RSV and parainfluenza by siRNA injected intranasally in mice, with or without transfection reagents (Bitko et al., 2005). In 2005 researchers targeted the RSV NS1 protein and showed that human dendritic cells transfected with siNS1 increased type-I interferons and induced the differentiation of naive CD4^+^ T cells into T helper type 1 (TH1) cells after RSV infection. They also showed that siNS1 nanoparticles may provide an effective inhibition of RSV infection in humans (Zhang et al., 2005). Three years later, the first clinical trial was conducted in lung transplant patients infected with RSV using siRNA. The siRNA designed for ALN-RSV01 is a 19-bp RNA duplex plus two (2′-deoxy) thymidine at both 3′ ends to prevent its nuclease degradation. The siRNA, ALN-RSV01, targets a highly conserved region in the RSV nucleocapsid (N) protein mRNA. ALN-RSV01 (NCT00658086) is a randomized, double-blind, placebo-controlled and multi-center trial which evaluated the safety and antiviral activity of vaccine in phase II of clinical trial. According to the latest update of NCT00658086, a phase II immunogenicity and safety of the candidate vaccine was performed in 24 participants who received RSV inoculation. ALN-RSV01 was administered daily by nasal spray, 2 days before and 3 days after RSV inoculation. Intranasal injection of ALN-RSV01 was safe and well tolerated and also showed a similar safety profile to saline placebo. Overall, ALN-RSV01 resulted in a 38% reduction in the number of infected individuals and a 95% increase in the number of uninfected individuals (DeVincenzo et al., 2010).

On the other hand, Khaitov et al. (2014) used siRNA to suppress allergen-induced responses for interleukin (IL)-4 and RSV P protein coding gene in BALB/c mouse model. Combined intranasal administration of anti-IL-4 and anti-RSV siRNAs resulted in a significant reduction of IL-4 mRNA and RSV viral RNA, and finally reduced eosinophils in bronchoalveolar lavage fluid and airway inflammation (Khaitov et al., 2014).

The authors investigated the M2-2 protein of RSV, which is important in the regulation of viral RNA transcription and replication. They designed siRNAs that specifically targeted the RSV M2-2 gene and determined their effectiveness at the protein (98%) and mRNA (83.1%) levels (Chin et al., 2016). According to these studies, it seems that important steps have been taken in the treatment of RSV based on siRNA, but more research is needed.

#### 5.2.2 Hepatitis C virus

Hepatitis C virus is a positive-sense, single-stranded, enveloped RNA virus belonging to the *Hepacivirus* genus of the Flaviviridae family. HCV is responsible for chronic liver diseases such as hepatocellular carcinoma and cirrhosis (Blanchard et al., 2002). This pathogen contains four structural proteins (Core, E1, E2, and P7) and six non-structural proteins (NS2, NS3, NS4A, NS4B, NS5A, and NS5B). Core, E1 and E2 are known as the main viral components of HCV particles. Meanwhile, P7 and NS2 are essential “cofactors” for virus assembly and NS3 to NS5B form a membrane-bound replicase complex (Jirasko et al., 2010). Currently, due to the high diversity of HCV strains, an effective treatment strategy for this virus infection has not been introduced. Several reports have shown the potential activity of siRNA against HCV, but it has not yet reached the clinical trial stage.

In 2003, Yokota et al. designed several siRNAs to target different parts of the HCV genome. They designed siRNAs to target the 5′ untranslated region (5′ UTR) of HCV genome, which resulted in 80% suppression of HCV replication with low concentrations (2.5 nM) of siRNA (Yokota et al., 2003). Subsequently, another study showed that the designed siRNA could inhibit HCV replication and expression of proteins (NS3-1948 and NS5B-6133) in Huh-7 cells stably replicating the HCV genome (Kapadia et al., 2003). These results indicate that RNAi can be a potential tool to help treat HCV in future studies.

Because HCV replicates in the cytoplasm of hepatocytes, RNA-based antiviral strategies are likely to successfully block the HCV replication cycle. Also, this molecule may inhibit cellular cofactors, such as proteasome a-subunit 7 (PSMA7) or Hu R antigen (HuR). Therefore, siRNAs have been shown to significantly reduce the levels of NS5B protein and HCV replicon RNA by silencing PSMA7 and HuR (Korf et al., 2005). A study transfecting the transcriptional plasmid DNA encoding the HCV 1a genome shown that three siRNAs targeting the E2, NS3, and NS5B regions effectively inhibited the expression of the core protein and the NS5A protein (Prabhu et al., 2005). On the other hand, it has been shown that siRNA targeted against NS5A of HCV genotype 1a inhibits the expression of NS5A and main protein in human hepatoma cells (HepG2) (Sen et al., 2003). In another method, the combination of RNAi mediated by lentiviral vector and IFN-alpha was used to evaluate HCV treatment and the results showed that IFN-α increases gene silencing and inhibits HCV proliferation (Pan et al., 2009). Several other studies have been conducted on the successful effect of siRNAs on non-structural proteins of HCV (Randall et al., 2003; Wilson et al., 2003; Kim et al., 2006; Liu et al., 2006).

Few studies have been conducted to evaluate the antiviral effect of anti-HCV siRNAs. In 2005, RNAi-mediated gene inhibition was first reported in an animal model after direct delivery of shRNAs. In this study, shRNAs against the conserved region of HCV internal ribosome entry site (IRES) were designed to measure their ability to inhibit HCV IRES-mediated reporter gene plasmid expression in human tissue culture cells and a mouse model. Finally, it was shown that specific shRNAs were effective in reducing the expression of luciferase-based on HCV IRES (Wang et al., 2005). Also, a designed siRNA targeting the NS5B region in a mice model reduced luciferase expression from a protein-luciferase fusion by 75% (McCaffrey et al., 2002). Kim et al., demonstrated the use of DTC-Apo consisting of cationic liposomes (DTC) and apolipoprotein A-I (apo A-I) with liver-specific siRNA delivery technology. They considered the potency and durability of gene silencing in mice after a single intravenous injection with DTC-Apo and showed that DTC Apo/HCV-specific siRNA administration inhibited viral gene expression by 65%–75% on the second day in the liver (Kim et al., 2009). Also, other studies showed the effect of siRNA against HCV in a mouse model for liver diseases (Pan et al., 2012).

One of the limitations of siRNA for target gene silencing is the lack of an efficient *in vivo* siRNA delivery system. The usage of the cationic lipid DOTAP was expanded in studies because it increases the complex formation with polyanionic nucleic acids such as siRNA and facilitates the interaction with the cell membrane. A cationic lipid-based anti-HCV (DOTAP) approach called nanosome was investigated along with several siRNAs targeting different sites of the HCV 5′-UTR. Meanwhile, systemic administration of combined siRNA-nanosomes in BALB/c mice is well tolerated without liver damage or tissue toxicity, and this indicates a significant reduction of HCV proliferation in a liver tumor-xenotransplant mouse model (Chandra et al., 2012). In addition, the evaluation of the effect of siRNA on protein kinase C-related kinase 2 (PRK2) was shown *in vivo*. PRK2 specifically phosphorylates NS5B by interacting with the N-terminal finger domain of NS5B and promotes HCV replication. Administration of PRK2 siRNA formulated with lipidoid nanoparticles (ND98 lipidoid, cholesterol, and PEG-ceramide C16) resulted in a decrease in serum HCV RNA titer, in the subcutaneous and orthotopic xenograft of mouse (Moon et al., 2016). Also, systemic administration of siPRK2 using galactosylated lipidoids caused more silencing of host PRK2 in mouse liver (≈80%) and faster suppression of HCV replication in HCV-xenograft mice (Park et al., 2016). The aim of all these studies is to show a promising path for siRNA-based HCV therapy in the future.

#### 5.2.3 Evaluation of siRNA in HBV preclinical and clinical studies

Hepatitis B virus is a hepatotropic virus with a partially double-stranded DNA genome of 3.2 kilobases (kb) (Washizaki et al., 2022). Proteins encoded by HBV include Core, pre-Core, Small (S), Middle S, Large S, Polymerase, and hepatitis B virus protein X (HBx) (Song et al., 2021). HBx, which is essential for the initiation and maintenance of replication, is considered the main cancer-associated protein in HBV infection (Zhao et al., 2021). This virus leads to chronic liver diseases such as chronic hepatitis, cirrhosis and hepatocellular carcinoma (McMahon, 2009). One of the promising therapeutic approaches that support the potential functional treatment of hepatitis B is siRNA (Wooddell et al., 2013; Huang et al., 2022). There have been several studies investigating the performance of RNAi in both preclinical and clinical studies (Table 4). The most important molecules in preclinical studies are ARC-520, ARB-1467, ARB-1740, and ALN-HBV.

**TABLE 4:** Clinical studies on siRNA in chronic hepatitis B.

Therapeutic drug name	Target	Delivery	Route of administration	Clinical trial number	Clinical trial stage	Status
ARC-520	X	GalNAc	IV	NCT02065336 NCT02738008	Phase II	Terminated
ARC-521	X and S	GalNAc	IV	NCT02797522	Phase I	Terminated
ARO-HBV	X and S	GalNAc	SC	NCT03365947	Phase I, II	Completed
ARB-1467	X and S	LNP	IV	NCT02631096	Phase II	Completed
ALN-HBV	X	GalNAc	SC	NCT02826018	Phase I	Terminated
IONIS-HBVRx	X	GalNAc	SC	NCT02981602	Phase II	Completed
DCR-HBVS	–	GalNAc	SC	NCT03772249	Phase I	Completed

A variety of viral and non-viral systems are being developed to deliver siRNA to the liver, tumors, and other tissues *in vivo*. In 2007, researchers were able to deliver siRNA to liver cells (both *in vitro* and *in vivo*), and the result was siRNA Dynamic PolyConjugates (DPC) (Rozema et al., 2007). Six years later, Wooddell et al. (2013) coinjection cholesterol-siRNA with N-acetylgalactosamine-conjugated melittin-like peptide (NAG-MLP) targeting liver cells, which was proposed as a promising treatment method for patients with HBV. The result of this coinjection was the suppression of multilogs of viral RNA and DNA, and proteins with a long duration of action (Wooddell et al., 2013).

Huang et al. (2022) showed significant results of ionizable liposomal siRNA in strong and continuous treatment of hepatitis B. In this study, a potent siRNA targeting HBV was selected and encapsulated with RBP131 with an approximate pKa value of 6.21 to construct a therapeutic formulation named RB-HBV008. They used mouse models (transient and transgenic) to investigate the effectiveness, and the results showed that the expression of viral RNAs and antigens (HBsAg and HBeAg), as well as viral DNA, was dose-dependent and time-dependent in the range of multilog reduction, both in the circulation and in liver tissue is suppressed (Huang et al., 2022).

The chronic agent of HBV, specifically the covalently closed circular DNA (cccDNA), is a highly stable and active nuclear episomal form of the viral genome that plays a key role in the viral life cycle. ARC-520 was the first RNAi therapy that included two siRNAs, cholesterol-siHBV74 and cholesterol-siHBV77, located at positions 118 and 71 bp upstream, respectively, and increased delivery of siRNAs to hepatocytes.

A randomized (phase I, NCT01872065), double-blind, placebo-controlled, single-center study was conducted in Melbourne (Australia) in 54 healthy volunteers (half male and half female) who received an intravenous dose of ARC-520 or placebo. Since the injection of ARC-520 is associated with the release of histamine, oral antihistamine treatment was recommended before starting the injection. The aim of this study was to evaluate parameters such as safety, tolerability, pharmacokinetics, and pharmacodynamics (Schluep et al., 2017). Further studies evaluated the effect of ARC-520 in combination with Entecavir for participants with hepatitis B surface antigen (HBsAg) in a clinical study (phase II, NCT02065336) in chronic HBV patients. The results showed that HBsAg decreased significantly in patients who were negative for HBV e antigen (HBeAg), while it decreased significantly in patients who were not HBeAg positive. On the other hand, ARC-520 reduced serum levels of HBsAg, HBeAg, and HBV DNA in chimpanzees. Arrowhead Pharmaceuticals recently conducted two multicenter, randomized, double-blind, placebo-controlled, multiple-dose phase II studies to evaluate levels of HBsAg reduction after intravenous administration of investigational product ARC-520 in a population of adults with CHB infection (Yuen et al., 2020). These two studies were stopped by the company decision.

Arrowhead Research Corporation was able to introduce another siRNA, ARC-521, into a phase I (NCT02797522) study in 47 participants. This intravenous combination was performed to evaluate the safety, tolerability, pharmacokinetics and antiviral activity in normal adult volunteers and patients with CHB, but a serious side effect was an increase in ALT up to 678 IU/ml as possibly related to the studied drug, it was recorded in the condition of non-adherence of nucleosid(t)e analogs (NA) (van den Berg et al., 2020). Overall, the ARC-520 and ARC-52 studies were discontinued due to lethal toxicity of the delivery formulation EX1 (a version of NAG-MLP).

Recently, a phase IIa clinical trial (NCT03365947) evaluated the efficacy of siRNA JNJ-73763989 (JNJ-3989) plus NA, with/without assembly encapsulant JNJ-56136379 (JNJ-6379, NCT03361956) in patients with to CHB. JNJ-3989 (formerly ARO-HBV), which is being developed in collaboration with Janssen Pharmaceuticals, is administered subcutaneously and is capable of targeting all HBV transcripts. JNJ-3989 was well tolerated in patients with CHB, reducing their HBsAg levels (<100 IU/ml in patients) and maintaining them for 336 days in 38% of patients after the last dose (Yuen et al., 2022).

Another siRNA, ARB1467, was evaluated in a phase IIa (NCT02631096) clinical trial by Arbutus Biopharma Corporation. ARB-1467, administered intravenously, targets viral RNA transcripts packaged within specific lipid nanoparticles (LNPs). Overall, treatment with this combination was well tolerated and no significant elevation of ALT was observed (except for one patient). In addition, the reduction of HBsAg after several doses was shown in HBeAg-negative and HBeAg-positive patients.

ALN-HBV is another combination of RNAi that is provided by Alnylam Pharmaceuticals in phase I/II. The compound, which has a subcutaneous injection, is planned to be studied in several directions: in healthy volunteers, a single ascending dose study and a multiple ascending dose study in CHB patients. The company discontinued development of ALN-HBV01 to advance a new development candidate, ALN-HBV02, which Enhanced Stabilization Chemistry-Plus (ESC+) GalNAc conjugate technology.

#### 5.2.4 *Ebola* virus

*Ebola* virus, a fatal hemorrhagic viral disease, is a negative-sense RNA virus belonging to the Filoviridae family (Yuan et al., 2022). In a preclinical study, LNP-encapsulated siRNAs designed to target *Ebola* virus were able to protect rhesus monkeys against this challenge (Thi et al., 2015). On the other hand, Tekmira (TKM-100802) has been evaluated in guinea pig, non-human primate (NHP), and human phase I clinical trials (Geisbert et al., 2010). TKM-Ebola consists of two siRNAs and targets three of the seven Ebola proteins (L, VP24, and VP35). Administration of TKM-100802 to patients infected with this virus (five patients) and one person as prevention after exposure showed that the efficacy or safety of this compound in the treatment of *Ebola* virus is not possible (Kraft et al., 2015; Liddell et al., 2015). Then a new formula of TKM-100802, TKM-130803 was presented. This new formulation included two nucleotide substitutions in siRNA VP35 and one nucleotide substitution in siLpol-2. Administration of TKM-130803 by intravenous infusion had no survival benefit (Kraft et al., 2015). However, according to the mentioned studies, siRNAs showed poor clinical results against the *Ebola* virus.

#### 5.2.5 Human immunodeficiency virus

Human immunodeficiency virus belongs to the genus *Lentivirus* and is grouped in the family Retroviridae, which causes acquired immunodeficiency syndrome (AIDS) (Baba et al., 2000). Two types of HIV have been identified: HIV type 1 (HIV-1) and HIV type 2 (HIV-2). HIV-1 is more dangerous and infectious than HIV-2. In 2002, for the first time, a study was conducted to treat HIV-1 infection using siRNAs. In this study, with designed siRNAs, they were able to 4 logs of inhibition of expression from the HIV-1 DNA (Lee et al., 2002). Two strategies can be defined to inhibit the replication of HIV through RNAi. The first is to target the structural genes *gag*, *pol*, and *env*, the regulatory genes *rev* and *tat*, and the accessory genes *vpu*, *nef*, *vpr*, and *vif*, and the second is to target the cellular genes required by HIV for replication (Bennasser et al., 2007; Bobbin et al., 2015). Several studies have been conducted in relation to the inhibition of HIV replication by siRNAs using cell culture methods, but only a few of them have reached the clinical trial level.

## 6 Criteria for designing any anti-cancer and anti-infection siRNA drugs

There are several criteria proposed for designing siRNA and siRNA delivery systems, especially in cancer therapy. SiRNA delivery is limited by their large size, which is sometimes up to 13 kDa and negatively charged because of phosphorylation at both 3′ ends which led to low bioavailability and weak penetration across the cancerous cell membranes (Whitehead et al., 2009). Formulation of siRNA with nanoparticles, polymers and protein or lipid-based systems, co-administration of siRNA with anticancer drugs as well as applying chemical modifications to the structure of siRNA are good solutions for the aforementioned limitations. Suitable chemical alterations leads to increase the siRNA stability and persistence in serum, a reduction in its side effects and better penetration into vascular barriers and tissues (Singh et al., 2018). Therefore, ideal siRNA delivery systems results in reduced interaction with normal body cells and serum proteins, specific delivery of siRNA to the target area compared to normal tissues, resistance to fast clearance and resistance to degradation by serum nucleases, lack of immunogenicity, while it must be degradable and compatible with the environment (Alexis et al., 2008; Whitehead et al., 2009; Singh et al., 2018). The criteria for the design of siRNAs are having 30%–52% G/C content, 3 “A/U” bases at positions 15–19, an “A” base at position 19, an “A” base at position 3, “U” base at position 10 in sense strand, absence of internal repeats and stability of sense and antisense strands (Reynolds et al., 2004). Thermodynamic properties of siRNA and accessibility of the target mRNA, as well as the availability of free ends of antisense siRNA are other factors that affect on the efficiency of siRNA (Kurreck, 2006). Off-targeting and immune stimulation also must be considered in drug specificity (Bumcrot et al., 2006; Zhang et al., 2021).

## 7 SiRNA administration strategies

**FIGURE 4:**
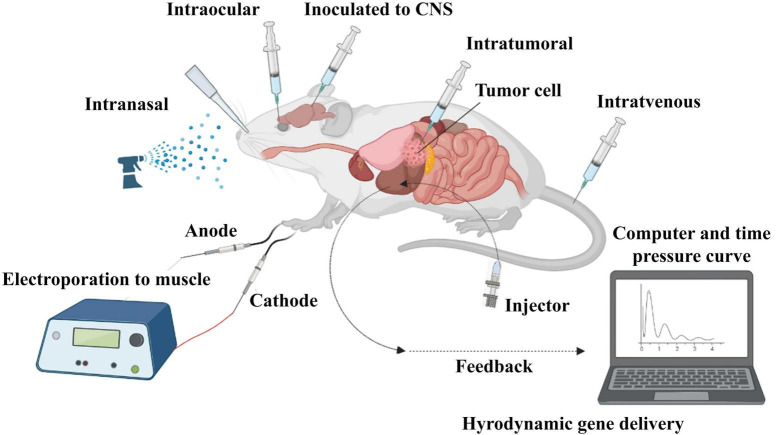
*In vivo* administration of siRNA. SiRNA was further evaluated in mouse models. Delivery routes are divided into local and systemic ways. Intravenous, intraperitoneal, intratumoral, intraocular, intranasal, intracerebral, and intramuscular are widely used for *in vivo* delivery of siRNA in studies.

There are two strategies for siRNA delivery based on route of administration; local and systemic methods. Local administration is used to treat diseases related to a specific organ of the body, such as the eye, lungs, skin, and oral cavity. Systemic administration is mostly used for the treatment of systemic diseases and metastatic cancer through nervous, gastrointestinal, and respiratory tracts (Figure 4; Chandela and Ueno, 2019). Reduced side effects and requiring a lower dose are the main advantages of local administration. Systemic administration is much more advanced than local administration, but it also has disadvantages, such as the degradation of siRNA in vessels by enzymes, endocytosis by other cells and their escaping, side effects, less specificity and faster rapid clearance in the body. Using nanocarriers improve systemic administration, which is more clinically used in blood-related diseases and cancers (Wang et al., 2010).

### 7.1 Local administration of siRNA

#### 7.1.1 Intraocular route

Ocular administration is one of the first ways of siRNA delivery due to the ease of access to the eye space and the safety profile (Gupta et al., 2019) which its effectiveness was reported in the treatment of blindness (Conley and Naash, 2010). For example, retinal destruction ganglion cell (RGC), which is followed by two important eye complications, is caused by the action of caspase-2 nuclease. The injection of IVT siRNA selectively prevents the expression of caspase-2 nuclease gene and prevents the occurrence of disorders in anterior ischemic optic neuropathy and glaucoma-related blindness in people. This type of siRNA transfer is not only safe and prevent eye inflammation, but also siRNA is able to stay in the eye for a longer time and exert its effects (Ahmed et al., 2011). Physiological barrier due to constant washing of the eye by the tear film and the impermeability of the epithelial cells of the cornea, conjunctiva, and existence of blood-retinal barrier are the main drawbacks of ocular administration that lead to a decrease in bioavailability and absorption of drug (Bodor and Buchwald, 2005; de la Fuente et al., 2010).

#### 7.1.2 Pulmonary route

Transfer of siRNA to the lungs through the pulmonary route is carried out in three ways: inhalation, intranasal route, and intra-tracheal administration. Pulmonary administration can protect the drugs against nucleases (Bodor and Buchwald, 2005). Local pulmonary administration is applied for treatment of bacterial and viral infections (mycobacterial infections and influenza), lung cancer, hypersensitivities, and respiratory fibrosis (pulmonary fibrosis) (Gupta et al., 2019). As an example, siRNA targeting RSV nucleocapsid synthesis and Na+ channel (ENaC) gene are applied for treatment of RSV upper respiratory tract infection and Cystic fibrosis (Khatri et al., 2012). The disadvantage of this administration is sensitivity to physiological barrier like mucus flow and respiratory cilia movement (Griesenbach et al., 2006; Gutbier et al., 2010).

#### 7.1.3 Administration to CNS

Generally, there are three ways to access the brain space: Intravenous, Intracerebroventricular, and Intranasal administration. Intravenous and intranasal delivery of siRNA to the CSF are non-invasive ways by which siRNA can reach the brain by passing through the blood–brain barrier (BBB). However, intranasal administration is not commonly used due to its limitations in the absorption of siRNA by the nasal epithelium. Intracerebroventricular also has access to the BBB (Nishina et al., 2013). Brown et al. showed that the combination of 2′-O-hexadecyl (C16) with fully modified siRNAs enables safe, potent and durable silencing in the CNS, eye and lung in rodents and non-human primates. C16-siRNAs delivered intrathecally or intracerebroventricularly were active across CNS regions and cell types with sustained RNAi activity for at least 3 months (Brown et al., 2022). IV administration of siRNA conjugated to nine-arginine-conjugated rabies virus glycoprotein peptide (RVG-9R) reaches the neuronal cells and significant GFP silencing in them (Kumar et al., 2007; Shyam et al., 2014).

For instance, increased beta-secretase (BACE1) activity is directly related to amyloid precursor protein (APP) and Alzheimer’s disease (AD). BACE1 activity produced amyloid-β (Aβ) from APP which pathologically Aβ amyloidosis involves in causing Alzheimer. So, siRNAs targeting BACE1 significantly decreased APP in transgenic mice for Alzheimer’s model (Singer et al., 2005). In a similar article, Alzheimer’s disease was studied by successful delivery of siRNA carried by nanoparticles to CNS which indicated the suppression of BACE1 and APP (2014). Amyotrophic lateral sclerosis (ALS) is another neurodegenerative disease which is induced by a mutation in gene encoding superoxide dismutase (SOD1). SiRNA delivery through a lentiviral vector into the mice model, showed survival of susceptible neurons by decrees in expression of SOD1 gene which led to more than 90% survival of the mice (Ralph et al., 2005). Huntington’s disease (HD) is a result of repeat expansion in polyglutamine which exacerbate function of huntingtin (htt) protein. The application of siRNA was successful in inhibition of *htt* gene and resolving irregularities induced by HD (Harper et al., 2005). Finally, Deng et al. (2005) evaluated the suppression effect of siRNA on PTEN-induced kinase 1 (PINK1), which mutation in this gene give rise to parkinsonism. The results showed 70% inhibition in PINK1 expression, activation of apoptosis and reduced durability of SH-SY5Y cell line (Deng et al., 2005).

#### 7.1.4 Intratumoral route

Considering siRNA can be also used as an anticancer agent, preliminary studies have shown that systemic administration of siRNA is effective for targeting cancer cell metastases. However, recent studies found that the potential of siRNA may decrease due to the breakdown by the kidney and liver during the systemic method. Otherwise, siRNAs can be administered directly to the tumor area in a complex with other molecules to be more protected, increase their uptake by cells, and no serious complications (Takahashi et al., 2005; Mu et al., 2009; Cao et al., 2019; Kanehira et al., 2019). Grabowska et al. (2015) delivered transferrin-conjugated branched PEI (TfPEI)-complexed siRNA by polycation polyethylene mine (PEI) directly to colonic and gastric gastrointestinal cancer cells by intratumoral administration which showed 43% silencing of the target gene. Another study, design a siRNA with an Arg-Gly-Asp (RGD) peptide-labeled chitosan nanoparticle (RGD-CH-NP) to target ovarian carcinoma in orthotropic mouse models. In addition to successfully intratumorally delivery of siRNA to the target cells, Han et al. (2010) also showed silencing in growth-related genes like POSTN, FAK, and PLXDC, and inhibition of tumor growth as well.

#### 7.1.5 Gastrointestinal route

Gastrointestinal (GI) transfer of siRNA is performing by endoscopic procedure. This route of administration is more considered for esophageal cancer, celiac and inflammatory bowel disease (IBD) by GI delivery of siRNA (Gupta et al., 2019). Carriers like lipid-based systems and macromolecules and siRNA chemical modifications are introduced to overcome the degradability of siRNA during passage in rough condition of GI tract before it can be able to affect the area (Chevalier, 2019). There is evidence which claimed siRNA may confer more effective results compared to surgery and chemotherapy as conventional therapy for esophageal malignancy. Effective apoptosis in esophageal cancer cell was observed following siRNA targeting anti-apoptotic *bcl-XL* gene (Gao and Huang, 2009). Another example is about overexpression of serine-arginine protein kinases (SRPK) and their involvement in adenocarcinoma. SRPK was significantly targeted by SRPK1-specific siRNA which led to limited permanence of cancerous cell in pancreatic and colon due to apoptotic death (Pellish et al., 2008).

#### 7.1.6 Electroporation to muscle

The use of high voltage for nucleic acid transfection into cells is called electroporation. This high voltage removes the cellular barriers for the passage of nucleic acids (Potter, 2003). Electroporation can be performed on any tissue. In this method, electrodes are placed around the area to be injected, then the corresponding position is subjected to high voltage. Gene transfer of siRNA by electroporation was investigated more in muscle and liver (Kishida et al., 2004). Golzio et al. (2005) in their study indicate good electrical transfer of the specific green fluorescent protein (EGFP) egfp22 siRNA to the muscles of adult mice by fluorescence imaging without any damage to mussel, which can reduce the expression of the cotransferred transgene. In another study by the same author, siRNA was able to reduce the size of melanoma tumor cells (Rols et al., 1998). All these studies describe the safety, reproducibility, specificity and at the same time simplicity of electropulsation for siRNA delivery.

#### 7.1.7 Vaginal route

Sexually transmitted diseases (STD) are also targeted by siRNA-based approach due to the lack of friendly prophylactic method for relief of patients suffering STD. For instance, treatment of HIV and HPV infections were tested through silencing E6 and E7 and (*gag*, *env*, *tat*, etc.) genes by siRNA can reduce the proliferation of cervical cancer cells and disrupt the cycle of HIV replication, respectively (Wu et al., 2011). However, physical changes like menstrual cycle, pH fluctuant and mucosal barrier may interfere with uptake and function of siRNA, which can be overcome by nontherapeutic, cell penetrating peptide (CPP) and macromolecule-based system for efficient delivery of siRNA (Rossi, 2009; Baxi et al., 2020). Among viral vectors, lentivirus- and adeno-based vectors are two popular types of vectors for intravaginal delivery, however, nowadays they are no longer used (Castanotto and Rossi, 2009) and substituted by novel non-viral vectors like lipid-based system and Aptamers.

## 8 Systemic administration of siRNA

### 8.1 Intravenous injection

Intravenous (IV) administration is an effective and fast method for systemic administration of drugs. However, the sensitivity of naked siRNAs to the hydrolyzing enzymes, rapid clearance of siRNAs from the bloodstream, and the aggregation of serum proteins are the main obstacles to IV administration. Kamlah et al. (2009) study showed IV injection of siRNA in Lewis lung carcinoma cancer model significantly targets hypoxia-inducible factor (HIF-1α and HIF-2α) and inhibited tumor cells growth. HIFs play an effective role in carcinogenesis through the hypoxic pathway and contribute to tumor survival, therefore the transcripts of HIFs can be considered to target (Jun et al., 2017).

### 8.2 Intraperitoneal injection

Intraperitoneal (IP) administration as another way for systemic administration is more effective in cancer and GI-related disease. IP injection conserve siRNA more than in IV route. Their effectiveness has also been shown in *in vivo* studies (Singhania et al., 2011). Inoue et al. (2008) showed apoptosis and destruction of cancer cells in drug-resistant gastric cancer patients following IP administration of nuclear factor-kappa B (NF-kB) p65 targeting siRNA with paclitaxel.

### 8.3 Oral route

Oral administration of gene therapy with acid nucleic-based drugs like siRNA, microRNA, DNA vaccines, etc., may be considered as an obstacle, but it also has advantages, such as easy administration and cost-effectiveness (O’Driscoll et al., 2019). Also, oral administration of siRNA is considered to be the best method of administration considering the comfort of the patient (Gupta et al., 2019). This method is more prefer to cure digestive-related disease like inflammatory bowel disease and gastrointestinal cancer because oral administration of siRNA directly to the damaged tissue. In comparison to other intestinal therapeutics, oral delivery of siRNA is less invasive and describe as patient friendly way of delivery of drug (Ball et al., 2018). Based on the preclinical studies, oral delivery of siRNA was successfully used to reduce the excess TNF-α levels to mediate the inflammation of intestinal tissue (Kriegel and Amiji, 2011). However, as mentioned earlier siRNAs were prone to degradation by enzymes in digestive, or their effectiveness may have been limited by acidic condition of stomach, which these hurdles are overcome by nanocarrier-based systems to have stable transmission (Akhtar, 2009; Gupta et al., 2019; O’Driscoll et al., 2019).

### 8.4 Viral and nonviral delivery systems

RNA-mediated therapy has been introduced as a promising and potential approach for the treatment of pathological conditions. So far, three categories of viral vectors, retroviruses (lentiviruses), adenoviruses and adeno-associated viruses (AAVs), and four categories of non-viral vectors, NPs, polymeric NPs, inorganic NPs, and biomimetic NPs are available (Yan et al., 2022). In this review, we introduce viral and non-viral vectors for gene delivery as well as recent and promising developments of these carriers in pre-clinic and clinical trials.

Nonviral vectors have been shown to have high biosafety, low cost, and easy production, as well as effective adjuvant activity that induces cellular immunity (Ramamoorth and Narvekar, 2015).

### 8.5 Viral vectors

Viral vectors encoding siRNAs or miRNAs have been used to trigger RNAi and gene silencing effects. Also, these vectors are an efficient means of gene transfer that are used today in academic research for research applications and clinical gene therapy (Warnock et al., 2011). Lentiviruses are a genus of the Retroviridae family that consists of an enveloped, single-stranded, positive-sense RNA sequence (Balvay et al., 2007). These viral vectors have several characteristics that make them suitable for therapeutic purposes for transgene delivery. Lentiviral vectors can deliver up to 9 kb of sequence and have the ability to express multiple genes from one vector (Bulcha et al., 2021). The effect of these vectors has been well studied in a wide range of neurodegenerative disorders, including Alzheimer’s disease, Parkinson’s disease, and HD (reviewed in Wong et al., 2006).

Adenovirus is a non-enveloped, double-stranded DNA virus that mainly causes upper respiratory tract infections, but can also infect other organs such as the brain and bladder (Bulcha et al., 2021). In recent years, there has been a great interest in the use of viral vectors, especially adenoviral vectors, in order to deliver therapeutic genes. These viral vectors have major advantages. First, they have high transduction efficiency for *in vivo* gene delivery. Second, while there are concerns about the safety of using adenovirus vectors, several clinical trials have evaluated the safest routes of administration. Third, these viral vectors offer strategies for developing strategies (to modify viral capsids) to increase therapeutic efficacy as well as improve virus targeting properties. Finally, the availability of scalable production systems is another advantage of this vector (Lee et al., 2017).

### 8.6 Adeno-associated virus

Adeno-associated virus is a non-enveloped virus belonging to the genus Dependoparvovirus of the Parvoviridae family. Long-term research led to the discovery of achievements of AAV in the configuration and composition of its genome and other features. These successes eventually led to the successful cloning of the wild-type AAV2 sequence into plasmids. For this reason, AAV was used as a gene delivery tool (Wang et al., 2019). Preclinical and clinical studies of AAV have gained popularity as an ideal therapeutic vector in aiding gene silencing and editing.

**FIGURE 5:**
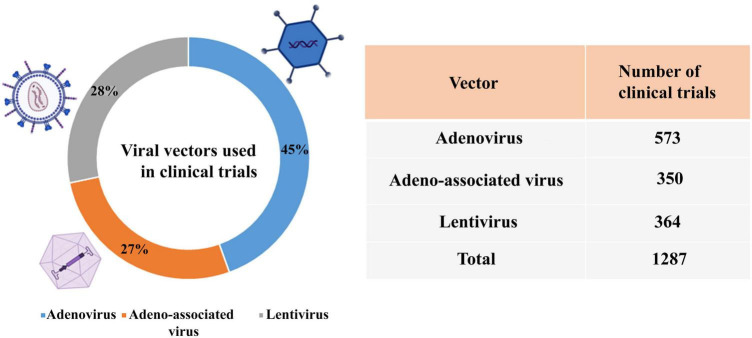
Viral vectors used in clinical trials.

According to the reports of the database,^3^ the percentage of viral vectors used in clinical trials is adenovirus (45%), lentivirus (28%), and AAV (27%) (Figure 5).

## 9 Advantages and disadvantages of viral vectors

The mentioned viral have low toxicity. Also, the common feature of these vectors is their long-term expression. Among the advantages of lentivirus, we can mention infecting both mitotic and non-mitotic cells and finally stable gene expression. On the other hand, the use of adenovirus vector infects both dividing cells and non-dividing cells (similar to AAV vector). In addition, adenovirus vectors are inserted without mutagenesis. The advantages of herpes virus vectors are their relatively long transgene expression and their safety for use in immunocompromised patients (Vannucci et al., 2013; Wold and Toth, 2013; Borel et al., 2014; Hutson et al., 2014; Zinn and Vandenberghe, 2014; Almarghalani et al., 2022).

There are concerns about insertional mutagenesis after integration of the viral genome into the lentivirus vector. According to studies, adenovirus and AAV vectors have dose-dependent hepatotoxicity and slow onset of gene expression, respectively. There is also high immunogenicity in viral vectors (especially in adenoviruses). In addition, herpes simplex vectors have shown cytopathic effects (Vannucci et al., 2013; Wold and Toth, 2013; Borel et al., 2014; Hendrickx et al., 2014; Hutson et al., 2014; Zinn and Vandenberghe, 2014; Almarghalani et al., 2022). Therefore, despite low transmission efficiency, non-viral vectors gained popularity due to better safety profile and lower production cost.

## 10 Non-viral vectors

### 10.1 Lipid-based nanoparticles

Nonviral vectors are more effective, low toxic, and potentially safer than viral vectors (Zhu et al., 2022). One of the most widely used non-viral gene carriers are lipid-based carriers that have a high potential for delivery of nucleic acids, including mRNAs. Lipid-based nanoparticles (LNPs) are mainly composed of ionizable cationic lipids, phospholipids, cholesterol, and/or poly (ethylene glycol) (PEG) lipids (reviewed in Yan et al., 2022).

Meanwhile, LNPs were used in Patisiran (ONPATTRO), the first approved siRNA drug, to treat transthyretin-mediated amyloidosis. In addition, the use of LNPs for antigen presentation and enhanced immune stimulation to elicit strong humoral and cellular immune responses led to the emergency approval of a COVID-19 vaccine (BioNTech/Pfizer and Modern) with the help of these carriers from the FDA.

In the 1970s, liposomes were introduced as a carrier for drug delivery. Liposomes (microscopic vesicles) are used to deliver a wide range of materials including hydrophilic or hydrophobic drugs, diagnostic agents, proteins, DNA and RNA (Gregoriadis and Florence, 1993).

### 10.2 N-acetylgalactosamine-siRNA conjugate

N-acetylgalactosamine (GalNAc) is a targeting ligand with high affinity and specificity for binding to the asialoglycoprotein receptor (ASGPR), which is mainly expressed in hepatocytes (approximately 10^6^ per hepatocytes) and results in rapid endocytosis (Springer and Dowdy, 2018). After binding, due to the drop in endosomal pH, GalNAc-siRNA conjugates are released from ASGPR into the endosomal lumen, and ASGPR returns to the hepatocyte surface. Then, GalNAc and linkers are rapidly degraded from the siRNA conjugate. Finally, through an unknown mechanism, a very small amount of siRNA (<1%) enters the cytoplasm and induces an RNAi response (Springer and Dowdy, 2018).

The reason for choosing ASGPR is its very efficient intracellular circulation with a suitable blood circulation speed (about 15 min) compared to other cell surface receptors (90 min) (Yamada et al., 2020). In general, GalNAc has advantages in delivery strategy over LNP. Among these advantages, the injection method (subcutaneous), dosage and frequency of administration can be mentioned more easily. Also, nowadays GalNAc-siRNA conjugates are used to solve the problem of delivering siRNA to liver cells (Springer and Dowdy, 2018). Of the five drugs approved by the FDA, four of them [(Patisiran (Alnylam), Givosiran (Alnylam), Lumasiran (Alnylam), Inclisiran (Alnylam and Novartis) and Vutrisiran (Alnylam)] use GalNAc conjugation technology to deliver siRNA and have significant effects in the treatment of liver-related diseases (Yan et al., 2022). The target organ and administration route of all five approved drugs are liver and subcutaneous (except Patisiran), respectively. In addition to the five drugs approved by the FDA, five other siRNA candidates (fitusiran, cosdosiran, nedosiran, tivanisiran, and teprasiran) are undergoing phase III clinical trials. The method of administration, targeting, medical indications related to these drugs are shown in Tables 1, 2.

### 10.3 FDA-approved RNAi-based drug and GalNAc-conjugated siRNAs

Patisiran (Onpattro^®^; Alnylam), the first RNAi-based drug, was approved by the FDA on 10 August 2018. This siRNA drug (active on the liver), an LNP siRNA, was recognized for the treatment of hTTR with polyneuropathy, which created a new revolution in the field of RNAi therapy (Adams et al., 2018). hTTR is an adult-onset, severe systemic, life-threatening disease caused by mutations in the gene encoding transthyretin (TTR). This disease is currently reported in at least 29 countries worldwide (including many European countries, the USA, China, and India) with genetic and phenotypic heterogeneity, characterized by multisystemic extracellular deposition of amyloid, leading to dysfunction of various organs and tissues (Adams et al., 2019). Alnylam continues to refine the potency of its GalNAc-conjugated siRNAs to improve clinical efficacy. So far, according to reports, approximately 75% of RNAi drugs in clinical trials are GalNAc-conjugated siRNAs (Zhu et al., 2022). Givosiran (GIVLAARI or ALN-AS1; Alnylam) is a GalNAc-conjugated siRNA drug approved by the FDA in November 2019 for the treatment of acute hepatic porphyria (AHP) (Agarwal et al., 2020; Scott, 2020). AHP is a rare genetic disorder based on mutations in genes involved in heme biosynthesis, which leads to the accumulation of toxic porphyrin molecules. Givosiran targets aminolevulinic acid synthase 1 (ALAS1) and can significantly reduce ALAS1 expression (Scott, 2020). Lumasiran (OXLUMO or ALN-GO1; Alnylam) is the third siRNsA drug approved by the FDA in November 2020 for the treatment of primary hyperoxaluria type 1 (PH1). PH1 is a rare genetic disorder that inhibits the expression of glycolate oxidase by targeting hydroxy acid oxidase 1 (HAO1). Lumasiran, like Givosiran, used the GalNAc-conjugated platform to achieve liver-specific delivery (Scott and Keam, 2021). Inclisiran (Leqvio^®^; Novartis) is another GalNAc-siRNA combination approved by the FDA in December 2021. Inclisiran targets the Proprotein Convertase Subtilisin/Kexin type 9 (PCSK9) gene and is known as the first siRNA drug to reduction low-density lipoprotein cholesterol (LDL-C) (Lamb, 2021). The successes of siRNA-based drugs were promising, so that the fifth drug, Vutrisiran (Alnylam), was approved by the FDA in June 2022. Vutrisiran is a double-stranded siRNA-GalNAc conjugate that targets TTR mRNA for the treatment of hTTR polyneuropathy (Keam, 2022).

### 10.4 Polymeric nanoparticles

Another one of the most widely used non-viral nanoparticles for gene delivery are polymers (Patil and Panyam, 2009). Due to the nature of flexibility, hydrophilicity, and neutrality, polymers can create surface barrier layers that reduce the amount of adhesion on the surface and finally make it invisible to invasive cells (Harris and Chess, 2003). *In vivo* studies investigated the potential effects of this nanoparticle in silencing the target gene. Meanwhile, it has been shown that polymeric nanoparticles made of polyamines can deliver siRNA to endothelial cells with high efficiency, thus facilitating simultaneous silencing of several endothelial genes *in vivo* (Dahlman et al., 2014).

### 10.5 Inorganic nanoparticles

Inorganic nanoparticles have been evaluated as a non-viral carrier in *in vitro* and *in vivo* studies, but no clinical trials have been observed so far (Sokolova and Epple, 2008; Ding et al., 2014; Zhang et al., 2014; Zhao et al., 2015; Luther et al., 2020). These nanoparticles have shown long shelf life, high stability (in a wide range of temperatures and pH), adjustable size and shape, high loading capacity and superior stability against enzymatic degradation. On the other hand, inorganic NPs have disadvantages such as poor biodegradability, limited efficiency and lack of clinical trials (Jiang et al., 2016).

### 10.6 Biomimetic nanoparticles

Another category of emerging drug delivery system is biomimetic nanoparticles, which have made significant progress in recent years (Beh et al., 2021). These nanoparticles have advantages such as high target specificity and long retention time and are usually made using endogenous substances (including cells, biomembranes, proteins, organelles, etc.), endogenous processes or special biological structures (Chen L. et al., 2021). On the other hand, endogenous processes have a wide spectrum, such as signal transduction or exosome-mediated material transport), antigen presentation, virus invasion, etc. Several studies have investigated the effect of using endogenous substances in siRNA for therapeutic purposes (reviewed in Yan et al., 2022). Finally, in 2018, an exosome-based siRNA delivery vector was evaluated in a clinical trial. This phase I trial (NCT03608631) studied mesenchymal stromal cell-derived exosomes with KrasG12D siRNA (iExosomes) to treat participants with pancreatic cancer with KrasG12D mutations that had spread to other parts of the body.

## 11 Limitations of siRNA application and how to get over them?

Small interfering RNA is designed to silence a certain gene, which makes them become a strong tool with antibacterial and antiviral properties (Gavrilov and Saltzman, 2012). Despite the beneficial effects of siRNAs, off-target effects, instability (by nucleases), challenges related to delivery and immune reactions have limited their application (Kang et al., 2023).

### 11.1 Off-target effects

Although siRNA is well known as a strong gene silencing tool, but it can cause non-specific off-target effects including immune response activation and changes in the non-target genes expression. Also, the effects of off-target gene knockdown which lead to reduction up to 1.5–4 time fold changes in the expression of various genes (Bridge et al., 2003; Jackson and Linsley, 2010).

The sequence similarities between siRNA and mRNA can be the reason for specific off-target effects, which occur when the 5′ end of the siRNA guide strand is complementary to the 3′ UTR of the mRNA. Therefore, the 5′ end sequence of the siRNA guide strand is important for silencing the off-target transcript. The competition of endogenous miRNAs with the foreign siRNAs that are transferred into cells and exposed to interference. It seems that gene regulation is disrupted in this state and causes unpredictable off-target effects. Other off-target effects of siRNAs can result in stimulation of innate immune responses to the oligonucleotides. SiRNAs have the ability to activate immune cells and induce the production of cytokines (Karikó et al., 2004; Hornung et al., 2005; Judge et al., 2005).

In general, one of the main challenges of siRNA therapy is to reduce off-target effects, as they can lead to cell death. On the other hand, low stability of the seed-target duplex reduces the ability of siRNA to induce seed-dependent off-target effects. Furthermore, both the duplex siRNA melting temperature of in a subset of the siRNA non-seed region and the GC content of its respective target sequences are associated with off-target reduction (Lam et al., 2015).

### 11.2 Chemical modifications

Based on studies, RNAs (single-stranded RNA compared to double-stranded RNA) are more sensitive to serum nucleases. In addition, abundant nucleases present in the bloodstream rapidly degrade naked RNAs in unmodified forms, which contributing to their short half-lives *in vivo* (Layzer et al., 2004). Therefore, chemical modification of RNA can be a potential help to optimize their efficiency.

Chemical modifications are necessary to provide stability, durability, better penetration into vascular barriers and tissues (Singh et al., 2018), and reduce off-target effects of siRNA in serum when administered *in vivo* (Kang et al., 2023). Chemical modifications commonly considered in siRNA design include: (a) modification of the 2′-OH ribose group, (b) locked and unlocked nucleic acids, and (c) phosphorothioate (PS) modification (Lam et al., 2015).

#### 11.2.1 Modification of the 2′-OH ribose group

Since the gene silencing activity of siRNAs does not depend on the 2′-OH ribose group, the modification of this agent has a potential impact on RNA duplex design (Chiu and Rana, 2003). Introducing the replacement of the 2′-OH ribose group with other chemical groups, including 2′-O-methyl (2′-OMe), 2′-methoxyethyl (2′-MOE), 2′-fluor (2′-F) can increase the stability of duplex RNA in serum. In addition, these substitutions (especially 2′-OMe) can play an important role in blocking endoribonucleases and produce a very strong modified siRNA (Jackson et al., 2006). Although a bulky substitution such as 2′-MOE may increase nuclease resistance, it is poorly tolerated in terms of activity (Prakash et al., 2005; Odadzic et al., 2008; Bramsen et al., 2009). On the other hand, Foster et al. (2018) showed that by further improving siRNA chemically, such as optimizing the modification position of 2′-deoxy-2′–fluoro (2′-F) and 2′-OMe on double-stranded siRNA, stability can be improved without affecting the intrinsic activity of RNAi to achieve significant therapeutic improvement. They used an iterative screening approach on multiple siRNAs to achieve improved designs with low 2′-deoxy-2′-fluoro content. The liver exposure data showed that the improvement in potency was mainly due to the increased metabolic stability of the siRNA conjugates. However, the good tolerance of siRNA containing 2′-F and 2′-OMe modifications has led to improved designs that achieve optimal results by adjusting the position and ratio of 2′-F and 2′-OMe in the two strands (Nair et al., 2017). In contrast, the introduction of 2′F substitution did not show proper activity, so that in the third stage of clinical trials Alnylam conjugate of N-acetylgalactosamine and siRNA containing 50% 2′F showed cardiotoxicity (Chernikov et al., 2019). In another study, it was shown that approaches such as blocking RISC siRNA loading with antisense 5′ caps, utilizing seed-pairing destabilization with GNA in the antisense strand, etc., reduced the hepatotoxicity observed with known toxic siRNAs, without changing the content of 2′F, 2′OMe, and PS or liver exposure (Janas et al., 2018).

Here, there are some examples where siRNA modification was investigated *in vivo* studies. Morrissey et al. applied chemical modifications including 2-fluoro, 2-O-methyl, and 2-deoxy sugars with phosphorothioate linkages in the siRNA structure targeting hepatitis B virus (HBV), and compared the result with unmodified siRNA in an animal model. They found that the modified siRNA remains stable in serum for a longer time, and the intensity of gene silencing increases in the same way. In their previous study, they evaluated chemically modified siRNA in HBV cell culture and effective gene silencing was observed (Morrissey et al., 2005). In another *in vivo* study, fully modified siRNA using 2′-O-methyl modifications and 5′ chemical stabilization by targeting sFLT1 against preeclampsia, also showed efficient gene silencing along with the safety (Davis et al., 2022). Tang et al designed fully chemically modified siRNA targeting human Janus kinase 1 (JAK1) with broad cross-species target ability against inflammatory and autoimmune skin diseases. By examining the optimized siRNA in the skin tissues of different species like human, pig, rat and, mouse, inhibition of JAK1 silencing gene (70%) was observed in the skin (Tang et al., 2024). Moreover, sometimes chemical modifications are applied to improve delivery of siRNA *in vivo*. For example, albumin-binding conjugate was tested for siRNA delivery, which establishing balance between hydrophobicity and safety in addition to not having interaction with blood components were reported in compare to other usual conjugate delivery (Fakih et al., 2023).

#### 11.2.2 Locked and unlocked nucleic acids

Another chemical modification is the introduction of locked nucleic acid (LNA), which can improve the stability of RNA duplex by increasing resistance to nuclease degradation (Elmén et al., 2005; Mook et al., 2007). On the other hand, numerous changes of this factor may lead to a decrease in efficiency in *in vitro* and *in vivo* conditions (Braasch et al., 2003; Grünweller et al., 2003; Elmén et al., 2005).

Unlocked nucleic acid (UNA), an acyclic analog of RNA that lacks the C2′ and C3′ links of the RNA ribose ring (Langkjaer et al., 2009). Although UNA modifications have improved performance and stability both *in vitro* and *in vivo*, but additional modifications can destabilize the duplex and reducing the melting point (Laursen et al., 2010).

#### 11.2.3 Phosphorothioate modification

In phosphorothioate (PS) modification, a non-bridging phosphate oxygen is replaced by sulfur (Campbell et al., 1990). This approach has been shown to effectively protect siRNA from degradation by exonucleases (Eckstein, 2014). Based on studies, modification of PS facilitates cellular uptake and bioavailability *in vivo* and improves the pharmacokinetics of nucleotides (Eckstein, 2014). In this regard, in 1998, this modification was successfully used in the drug Vitravene (fomivirsen) for topical delivery to the eye (Crooke, 1998). On the other hand, several studies reported that this modification caused increased toxicity and decreased gene silencing, and in the end, it was not very popular (Amarzguioui et al., 2003; Chiu and Rana, 2003; Braasch et al., 2004).

Another method, boranophosphate (BP) modification, replaces a non-bridging phosphodiester oxygen with an isoelectronic moiety of borane (BH3) (Hall et al., 2004). Hall et al showed that BP-modified ds-siRNAs were more active than PS for target gene silencing (Hall et al., 2004). In addition, BP is more resistant to nuclease and less toxic than PS (Chernikov et al., 2019). However, this modification requires more studies to improve the efficiency of siRNA in the treatment of diseases.

Although chemical modification helps target gene silencing and siRNA stability, but developing an effective tool for siRNA delivery is still a major challenge. In the previous sections (viral and nonviral delivery systems), different delivery system approaches have been discussed. The researchers showed that increasing its molecular weight by binding ligands, incorporating larger particles, or binding to plasma proteins effectively saves siRNA from elimination (Crooke, 1998).

### 11.3 Immunogenicity

Since siRNAs induce immune responses in both sequence-independent and sequence-dependent ways, strategies can be developed to prevent immune activation associated with this molecule (Kleinman et al., 2008; Meng and Lu, 2017).

Several immunostimulatory motifs that should be avoided have been reported, including 5′-UGU-3′, 5′-UGUGU-3′ and 5′-GUCCUUCAA-3′ (Judge et al., 2005; Fedorov et al., 2006). In addition, the presence of U-rich sequences with TLR 7/8 activation is related (Goodchild et al., 2009). Also, less immunostimulating vectors should be selected for siRNA delivery (Meng and Lu, 2017). Since siRNAs with more than 30 nucleotides can stimulate the immune response by activating the IFN pathway, therefore, reducing the number of siRNAs can reduce RNAi-based immune activation (Gantier and Williams, 2007; Meng and Lu, 2017).

Several endosomal TLRs like TLR3, TLR7, and TLR8 are involved in the recognition and response to siRNAs (Shi and Sun, 2018). These receptors induce interferons, tumor necrosis factor-alpha (TNFα), and interleukin-6 (Il-6) production. On the other hand, these double-stranded molecules stimulate monocytes and myeloid dendritic cells through TLR8 to produce pro-inflammatory cytokines. Also, TLR7 has the ability to stimulate plasmacytoid dendritic cells to produce interferon-α (IFNα).

## 12 Conclusion and outlook

Today, in the world of therapy, the discovery of siRNA as new class of therapeutic agents by silencing the gene(s) of interest has shown significant progress. So that during the last two decades, siRNA has been used in the treatment of various human diseases including cancers, viral and bacterial infections, eye diseases, genetic disorders, and cardiovascular diseases. Five drugs have been approved by the FDA over the past few years including Patisiran, Givosiran, Inclisiran, Lumasiran, and Vutrisiran. In addition, in the last two decades, tremendous progress has been made in the design and synthesis of vectors (viral and non-viral) for gene delivery, which have had promising results in studies. However, several developments are needed to achieve their full potential, requiring close collaboration between chemists, pharmacologists and biologists.

There is no doubt that siRNA has an effective potential in the treatment of a wide range of diseases. Considering the significant progress of studies in the development of treatment and also the approval of five drugs (Patisiran, Givosiran, Inclisiran, Lumasiran, and Vutrisiran) based on siRNA, we also are treated with the help of this silencer. On the other hand, there are some concerns for examining any new treatment method. Therefore, the long-term safety of siRNA is still unknown. Non-viral vectors are potentially safer than viral vectors, but studies of both vectors have shown significant improvements in target gene delivery.

In addition to the successes of the GalNAc-siRNA combination for hepatic delivery of siRNA, researchers have made significant progress for targeted delivery of renal, CNS, and ocular siRNA. Finally, due to the high number of clinical trial studies, lungs are thought to be a promising tissue for local delivery of naked siRNA, which could be promising for their siRNA-based therapy in the near future.

## Author contributions

RA: Writing – original draft, Writing – review & editing. HM: Writing – original draft, Writing – review & editing. MA: Writing – original draft, Writing – review & editing. AA: Writing – original draft, Writing – review & editing.

## Footnotes

1
https://clinicaltrials.gov/ct2/show/NCT03903393

2
https://clinicaltrials.gov/ct2/show/NCT03830619

3
https://a873679.fmphost.com/fmi/webd/GTCT
